# Selumetinib for pediatric arteriovenous malformation with a *MAP2K1* variant identified by liquid biopsy of nidus-draining venous blood

**DOI:** 10.1016/j.jdcr.2026.08.040

**Published:** 2026-09-02

**Authors:** Daichi Hayashi, Michio Ozeki, Akifumi Nozawa, Shiho Yasue, Saori Endo, Hiroshi Kawada, Masayuki Matsuo, Toshiaki Ishida, Hidenori Ohnishi

**Affiliations:** a Department of Pediatrics, Graduate School of Medicine, Gifu University, Gifu, Japan; b Laboratory of Intractable and Rare Diseases, Graduate School of Medicine, Gifu University, Gifu, Japan; c Department of Advanced Medicine for Refractory Vascular Anomalies, Graduate School of Medicine, Gifu University, Gifu, Japan; d Department of Radiology, Gifu University, Gifu, Japan; e Department of Hematology and Oncology, Hyogo Prefectural Kobe Children's Hospital, Hyogo, Japan; f Clinical Genetics Center, Gifu University Hospital, Gifu, Japan

**Keywords:** arteriovenous malformation, liquid biopsy, MAP2K1, MEK inhibitor, selumetinib

## Introduction

Extracranial arteriovenous malformations (AVMs) are rare, progressive vascular anomalies characterized by abnormal connections between arteries and veins without an intervening capillary network. Somatic gain-of-function mutations in the RAS/MAPK signaling pathway are a major cause of extracranial AVMs.^1^ An integrated analysis of 4 genetic studies encompassing 101 AVM patients demonstrated that *MAP2K1* was the most frequently mutated gene, accounting for 40.6% of cases.^2^

Mitogen-activated protein kinase kinase (MEK) inhibitors have emerged as a promising targeted therapy for AVMs harboring RAS/MAPK pathway mutations.^3^ In a multicenter prospective trial, trametinib produced significant hemodynamic improvement in patients with somatic *KRAS*/*MAP2K1* mutations.^4^ However, initiation of targeted therapy requires prior molecular diagnosis, which currently depends on tissue biopsy. In pediatric AVMs, direct open biopsy may be unfeasible because of the risk of bleeding and anatomic constraints. Therefore, cell-free DNA (cfDNA) analysis has been explored as a less invasive diagnostic alternative for vascular malformations.^5^ We report a pediatric case of extracranial AVM in which liquid biopsy using cfDNA from nidus-draining venous blood identified a *MAP2K1* variant, presumed somatic, enabling the initiation of MEK inhibitor therapy.

## Case report

A 2-year-old girl presented with progressive redness and swelling of the left forefoot, noted at birth (Fig 1, *A* and *B*). At the referring hospital, infantile hemangioma was initially suspected, and oral propranolol was started. The lesion continued to enlarge despite treatment (Fig 1, *C*). A vascular tumor was subsequently considered, and sirolimus was started without a tissue biopsy. However, the lesion did not improve and became increasingly painful (Fig 1, *D*). She was referred to our department at 2 years of age for further evaluation.

**Fig 1:**
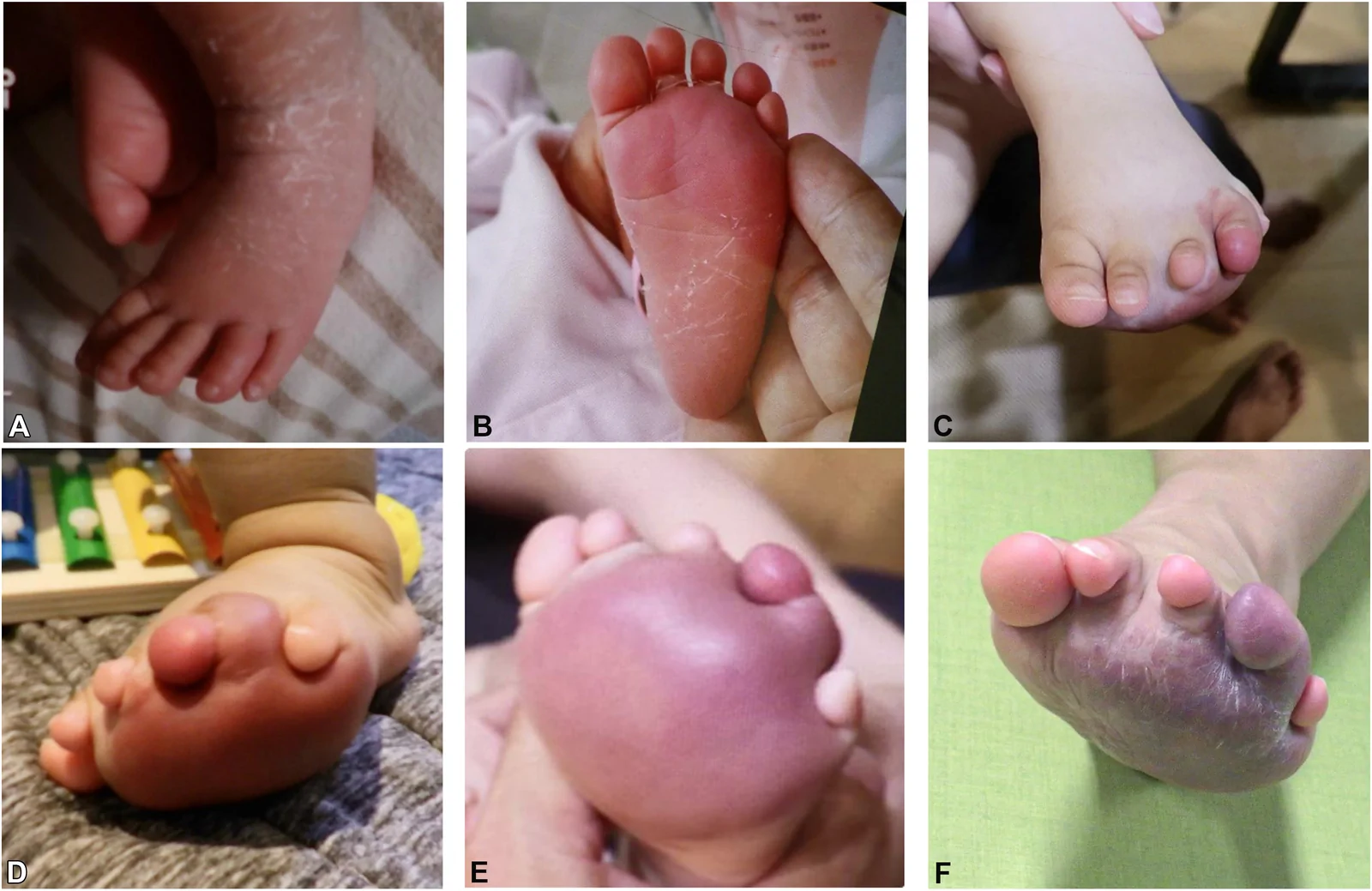
Clinical photographs showing the progression of the left forefoot lesion and treatment response. At birth, the lesion presented as erythema with mild swelling **(A, B)**. The swelling progressively increased at 1 year **(C)** and 1.5 years of age **(D)**. At 2 years of age, the forefoot showed marked swelling with tenderness on palpation **(E)**. Four months after the initiation of selumetinib therapy, the swelling was markedly decreased with skin wrinkling visible, indicating a reduction in lesion volume **(F)**.

Physical examination revealed a diffusely swollen, warm, erythematous left forefoot with a palpable thrill (Fig 1, *E*). Magnetic resonance imaging demonstrated a heterogeneous enhancing mass with flow voids (Fig 2, *A*), and digital subtraction angiography confirmed a nidus-type arteriovenous shunt with early venous filling (Fig 3, *A* and *B*). The lesion was diagnosed as a high-flow AVM classified as Schöbinger stage III based on progressive enlargement and refractory pain. Tissue biopsy was deemed unfeasible because of the high-flow nature of the lesion and hemorrhagic risk.

**Fig 2:**
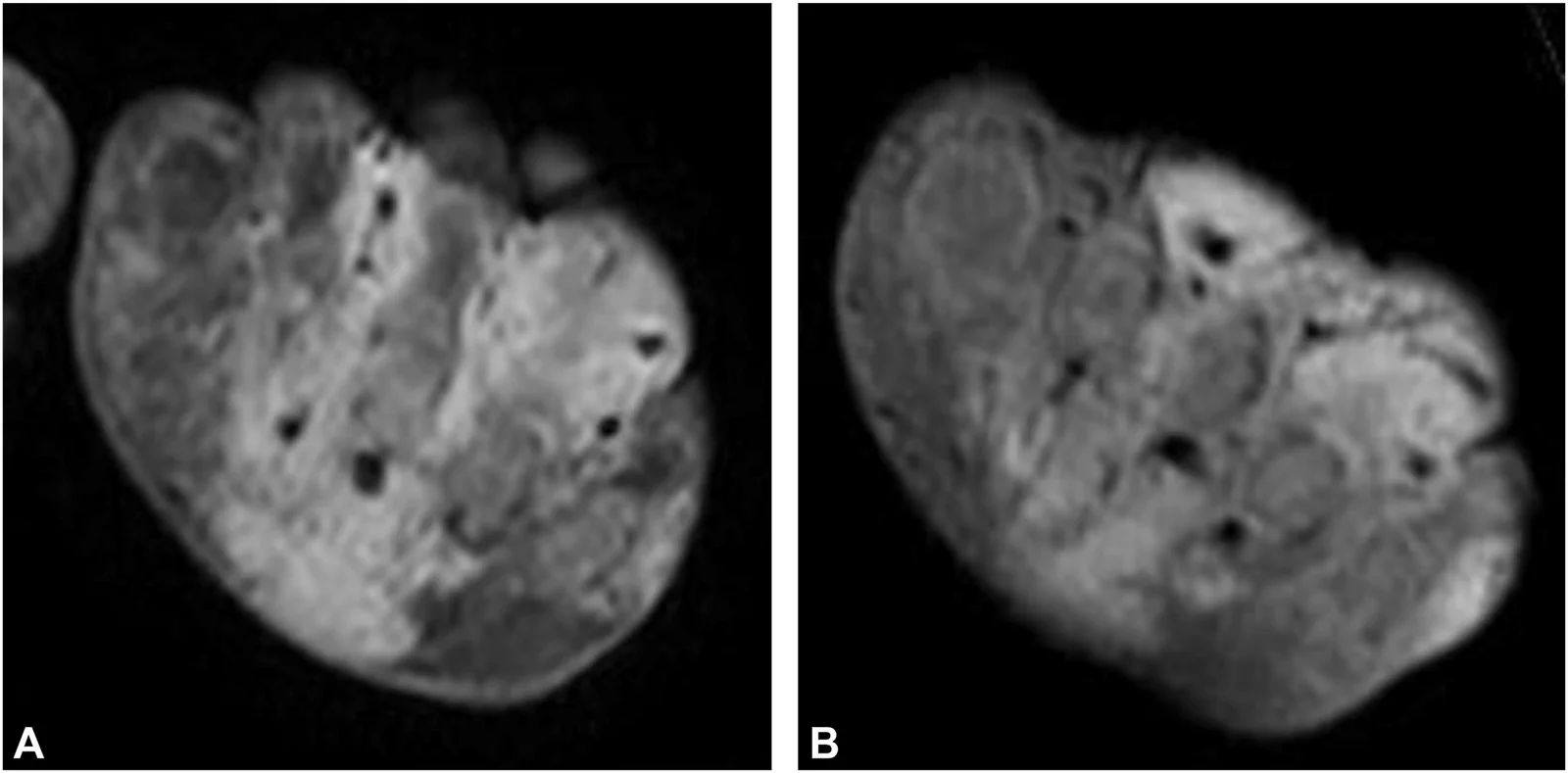
Contrast-enhanced magnetic resonance imaging of the left foot. Fat-suppressed postcontrast T1-weighted image in the axial plane at diagnosis demonstrates an enhancing soft-tissue mass with flow voids extending from the left toes to the plantar aspect of the foot, consistent with an arteriovenous malformation **(A)**. Four months after the initiation of selumetinib therapy, reduced enhancement of the lesion is seen **(B)**. Lesion volume was measured by multiplying the region-of-interest areas by the slice width using a Digital Imaging and Communications in Medicine viewer (OsiriX; Pixmeo, Bernex, Switzerland), as previously described.^6^

**Fig 3:**
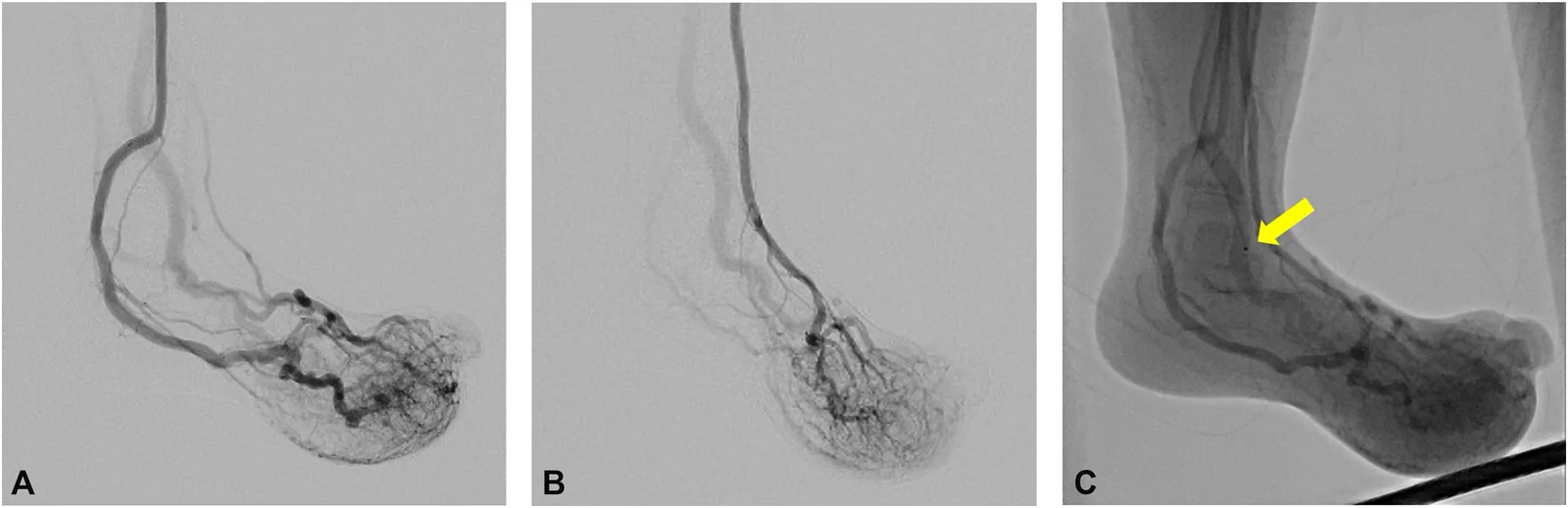
Digital subtraction angiography of the left lower extremity. Posterior tibial artery and **(A)** anterior tibial artery **(B)** injections demonstrate the arteriovenous malformation nidus in the forefoot with early venous filling. **C,** Popliteal artery injection with simultaneous venous access. A microcatheter introduced via the superficial femoral vein was advanced to the ankle, with its tip positioned within the great saphenous vein (*arrowhead*). The great saphenous vein is opacified by contrast during the arterial injection, confirming its role as a draining vein of the nidus. Whole blood was aspirated at this site for cell-free DNA analysis.

To obtain a molecular diagnosis, blood was collected from a nidus-draining vein under fluoroscopic guidance. A microcatheter was introduced via the superficial femoral vein and advanced to the ankle, where its tip was positioned within the great saphenous vein draining the AVM nidus (Figure 3, *C*). Whole blood (20 mL) was aspirated at this site, and cfDNA was extracted from plasma separated by centrifugation (3000 × *g*, 15 min, 4 °C). Targeted sequencing was performed using the TruSight Oncology 500 ctDNA v2 panel (Illumina) on a NovaSeq X platform (mean target coverage 3976 × ; indel limit of detection 0.5% variant allele frequency [VAF]). A *MAP2K1* c.171_185del (p.Gln58_Glu62del) variant was detected at a VAF of 2.6%. Paired germline testing was not performed; the variant was presumed somatic based on its established association with extracranial AVMs.^1^

Based on the identified *MAP2K1* variant, off-label use of the MEK inhibitor selumetinib was approved by the Institutional Review Board of Gifu University Hospital (no. 79). After parental informed consent, oral selumetinib (Koselugo 5 mg granules) was initiated (10 mg twice daily [20 mg/day; approximately 25 mg/m^2^/dose]). Within 4 weeks, the patient demonstrated clinical improvement with reduced lesional swelling and firmness, and pain relief. At the 4-month follow-up, magnetic resonance imaging showed reduced enhancement (Fig 2, *B*) with lesion volume decreasing from 56.3 to 30.1 cm^3^ (46.5% reduction), and clinical improvement (Fig 1, *F*) was sustained through the 6-month follow-up. Transient grade 1 diarrhea occurred during the first 2 months and resolved spontaneously; no other adverse events were observed.

## Discussion

The *MAP2K1* c.171_185del (p.Gln58_Glu62del) variant identified in our patient is an in-frame deletion within the negative regulatory domain of MEK1. Couto et al first identified somatic *MAP2K1* variants in extracranial AVMs and demonstrated that these mutations constitutively increased MEK1 activity.^1^ Schmidt et al confirmed the pathogenic significance of negative regulatory domain variants in a multicenter cohort.^7^ Notably, AVMs with *MAP2K1* mutations had a lower post-treatment progression rate than *RAS*-mutant AVMs (20.0% vs 76.9%),^7^ suggesting that genotype may influence prognosis and treatment response. Although histopathology was unavailable, the diagnosis was supported by absent consumptive coagulopathy, arteriovenous shunting indicating a high-flow malformation rather than a vascular tumor, and a *MAP2K1* variant reported in extracranial AVM but not kaposiform hemangioendothelioma.

A multicenter prospective trial of trametinib demonstrated significant hemodynamic improvement, with greater clinical responses in somatic *KRAS*/*MAP2K1*-mutants than in germline-variant AVMs.^4^ Selumetinib, an oral selective allosteric MEK1/2 inhibitor, directly targets MEK1, of which the *MAP2K1* variant in our patient was constitutively activated. Its pediatric safety profile was reported in the SPRINT trial for neurofibromatosis type 1–associated plexiform neurofibromas.^8^ To the best of our knowledge, selumetinib has not been previously reported for the treatment of extracranial AVM. Our patient’s response suggests that the benefit of MEK inhibition in *RAS/MAPK*-mutant AVMs^3^ may extend across MEK inhibitors. However, its long-term efficacy and safety remain unestablished, as evidence is limited to short-term observation.^4^

cfDNA liquid biopsy enabled molecular diagnosis in our case, where tissue biopsy was unfeasible. Zenner et al detected *MAP2K1* variants in plasma cfDNA from 2 of 8 AVM patients (VAF 0.4% to 2.1%),^5^ and our group previously demonstrated the cfDNA-based detection of *NRAS* variants in kaposiform lymphangiomatosis.^9^ However, the reported plasma cfDNA detection rate for AVMs remains limited (25%),^5^ partly because the detection of low-frequency mosaic variants requires highly sensitive sequencing approaches.^10^ In our case, blood from the nidus-draining vein may have enriched lesion-derived cfDNA, yielding a VAF of 2.6%, which exceeds the range reported by Zenner et al.^5^ Sampling blood from a nidus-draining vein during diagnostic angiography offers a practical strategy when tissue biopsy is unfeasible.

This report had several limitations: it describes a single case with a 6-month follow-up requiring validation in larger series, and the optimal cfDNA sampling conditions for AVM molecular diagnosis require further investigation.

In conclusion, the liquid biopsy of nidus-draining venous blood enabled molecular-targeted MEK inhibition with selumetinib in a pediatric extracranial AVM where tissue biopsy was unfeasible, warranting further investigation.

### Declaration of generative AI and AI-assisted technologies in the writing process

During the preparation of this manuscript, the authors used Claude (Anthropic) for language editing and manuscript formatting assistance. The authors reviewed and edited the output and take full responsibility for the content of the publication.

## Conflicts of interest

None disclosed.
